# Investigating the association between blood cobalt and gallstones: a cross-sectional study utilizing NHANES data

**DOI:** 10.3389/fpubh.2024.1363815

**Published:** 2024-02-07

**Authors:** Yunfan Li, He Han, Kuanxuan You, Chaojun Ma, Xin Fan

**Affiliations:** Department of General Surgery, Affiliated Hospital of Jiangsu University, Zhenjiang, China

**Keywords:** gallstones, blood cobalt, metal element, cobalt exposure, NHANES

## Abstract

**Background:**

With the use of cobalt alloys in medical prosthetics, the risk of cobalt exposure has increased. The objective of this study was to investigate the correlation between blood cobalt levels and the occurrence of gallstones utilizing data from the National Health and Nutrition Examination Survey (NHANES).

**Methods:**

Data collected between 2017 and 2020 were analyzed, encompassing a total of 5,610 participants. Cobalt concentrations in whole blood specimens were directly measured using inductively coupled plasma mass spectrometry (ICP-MS). The presence of gallstones was ascertained through a standardized questionnaire. To assess the association between blood cobalt levels and the presence of gallstones, logistic regression analysis, restricted cubic spline analysis, and subgroup analysis were utilized.

**Results:**

The results of logistic regression analysis revealed a heightened risk of developing gallstones in the Quartiles 2 and Quartiles 4 groups based on blood cobalt levels when compared to the Quartiles 1 group (OR = 1.54, 95% CI: 1.15–2.07; OR = 1.35, 95% CI: 1.03–1.77). The restricted cubic spline analysis exhibited a positive linear correlation between blood cobalt levels and the occurrence of gallstones. Subgroup analyses further demonstrated a statistically significant correlation between the Quartiles 4 category of blood cobalt levels and an elevated risk of gallstones, particularly among individuals aged 60 years or older, females, those with a body mass index (BMI) equal to or exceeding 25, serum total cholesterol levels below 200 mg/dL, as well as individuals diagnosed with hypertension or diabetes.

**Conclusion:**

Our study findings indicate a notable association between elevated blood cobalt levels and an increased risk of gallstones. To establish a causal relationship between blood cobalt levels and the elevated risk of developing gallstones, further prospective cohort studies are warranted.

## 1 Introduction

Gallstones are a prevalent digestive disorder characterized by the formation of stones in the gallbladder or bile ducts, primarily attributed to abnormally elevated levels of cholesterol or bilirubin in the bile. Globally, gallstone prevalence affects approximately 10–20% of adults, imposing considerable economic burdens on individuals and societies (1–4). Approximately 80% of gallstone patients remain asymptomatic; however, without timely intervention, the clinical course of gallstones can progress from asymptomatic carriers to symptomatic and complex conditions such as acute cholecystitis, cholangitis, pancreatitis, and, in rare instances, intestinal obstruction (5, 6). Numerous studies have demonstrated that disorders of cholesterol metabolism, unhealthy lifestyle habits, genetic factors, obesity, and pregnancy significantly contribute to the development of gallstone disease (1, 7–9).

Cobalt is an essential trace element that plays a crucial role in growth, development, maintenance, and overall health. It serves vital physiological functions through its involvement in vitamin B12 synthesis and coenzyme activity. Insufficient cobalt levels can result in anemia and hypothyroidism, while excessive cobalt exposure may lead to peripheral neuropathy, vision loss, sensorineural hearing loss, and cognitive decline (10–13). In the field of medical prosthesis implantation, cobalt-containing alloys are widely utilized due to their exceptional properties such as wear resistance, corrosion resistance, high mechanical strength, hardness, and fatigue resistance. However, these alloys also pose a risk for endogenous cobalt exposure (14).

Previous research has suggested a potential association between certain metallic elements, including selenium, cadmium, mercury, lead, and manganese in the bloodstream, and the development of gallstones. Specifically, some researchers have hypothesized that elevated selenium levels in the blood may serve as a risk factor for gallstone formation (15). However, upon reviewing relevant data, we found no studies that have investigated the potential connection between blood cobalt levels and the occurrence of gallstones. Consequently, this study aimed to examine the association between blood cobalt levels and gallstones in the U.S. population by analyzing the blood cobalt levels of patients diagnosed with gallstones based on data from the 2017–2020 NHANES. By doing so, it aimed to contribute additional evidence toward comprehending the prevalence and advancement of gallstone-related conditions.

## 2 Methods

### 2.1 Study design and participants

**Figure 1:**
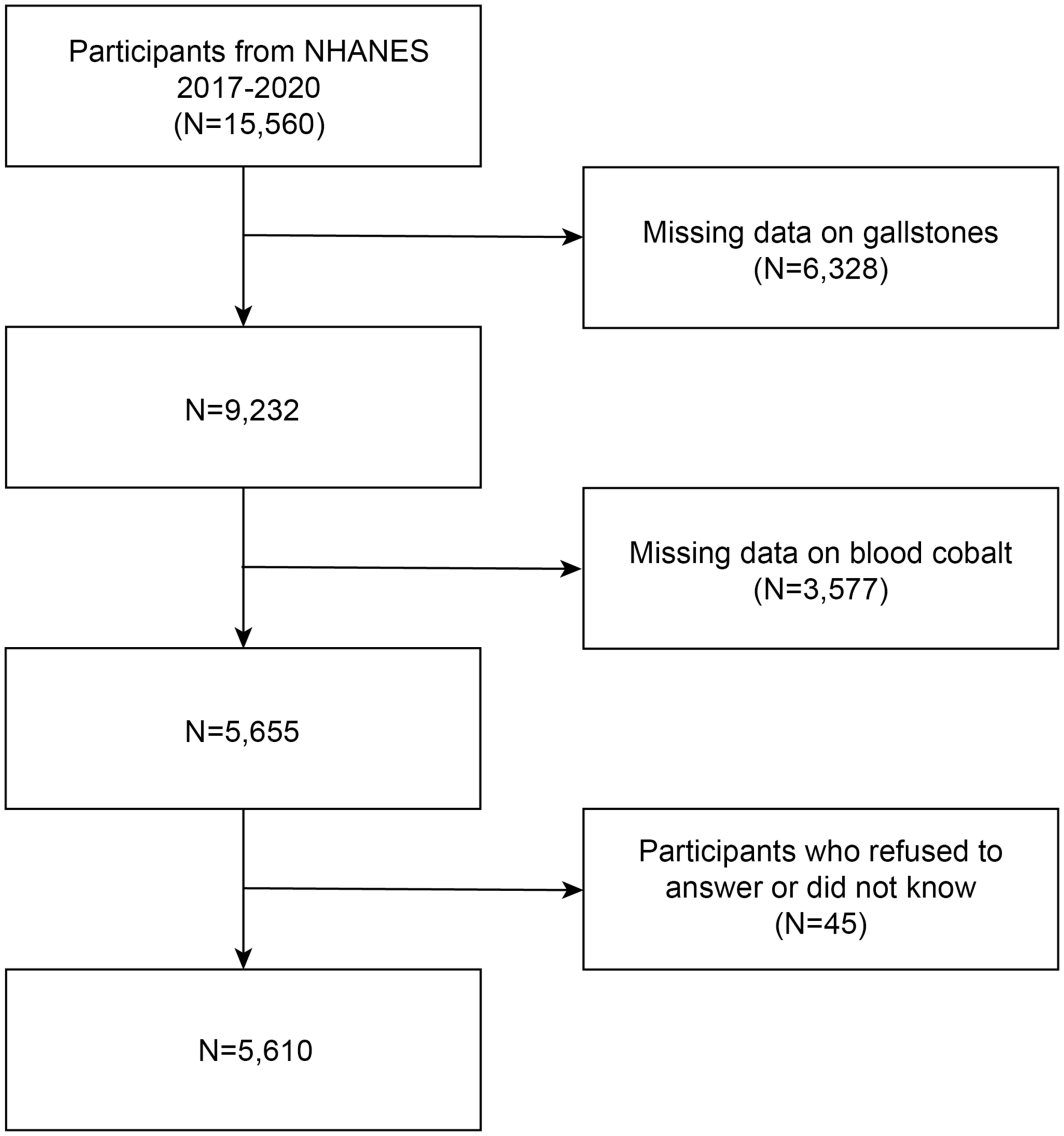
Flowchart of the study. NHANES 2017–2020 enrolled 15,560 participants. Of these, 5,610 were retained after fulfilling inclusion and exclusion criteria.

The National Health and Nutrition Examination Survey (NHANES) is a recurrent survey overseen by the National Center for Health Statistics (NCHS). It is a comprehensive, nationwide survey that aims to evaluate the health and nutritional status of individuals across all age groups in the United States. This population-based survey is specifically designed to provide representative data on adults and children throughout the country. The survey utilizes a comprehensive methodology that encompasses health interviews administered at respondents’ residences, health measurements obtained at mobile health check-up centers, and an extensive collection of demographic data, physical examinations, laboratory tests, health-related questionnaires, and prescription medication records. This multifaceted approach ensures a thorough assessment of various aspects related to health and allows for a comprehensive understanding of the participants’ well-being. The NHANES program as a whole has obtained approval from the Ethical Review Board of the National Center for Health Statistics, and all participants have willingly provided informed consent. Data from NHANES are released every 2 years; however, due to the 2019 coronavirus (COVID-19) pandemic, the program was temporarily suspended in March 2020. Consequently, data collected from 2019 to March 2020 were merged with the NHANES 2017–2018 cycle to form a nationally representative sample comprising NHANES 2017-March 2020 pre-pandemic data. Within this study cycle, information regarding the history of gallstones was specifically requested. Initially, 15,560 participants were enrolled; however, 6,328 participants did not complete the gallstone questionnaire, 3,577 participants had missing blood cobalt data, and 45 participants either declined to answer or indicated uncertainty in response to the gallstone, hypertension, or diabetes questionnaires. Consequently, these individuals were excluded from the study. The final analysis included a total of 5,610 participants, and a flow chart depicting the selection process is provided in Figure 1.

### 2.2 Definition of gallstones

To ascertain the presence or absence of gallstones, we employed a questionnaire titled “Has DR ever said you have gallstones” Participants responding affirmatively were classified as having gallstones, while those who responded negatively were categorized as not having gallstones.

### 2.3 Measurement of blood cobalt

Whole blood specimens were processed, stored, and subsequently sent to the Division of Laboratory Sciences at the National Center for Environmental Health, Centers for Disease Control and Prevention in Atlanta, GA for analysis. The concentrations of cobalt in these specimens were directly measured using inductively coupled plasma mass spectrometry (ICP-MS). The lower limit of detection (LLOD) for cobalt was determined to be 0.06 μg/L. If values fell below the LLOD during analysis, they were replaced with LLOD divided by √2.

### 2.4 Identification of covariates

The statistical model utilized the following variables as covariates: age, gender, race, education level, waist circumference, body mass index (BMI), ratio of family income to poverty, serum total cholesterol level, blood selenium level, serum iron level, and history of diabetes and hypertension. We employed the questionnaire “Doctor told you have diabetes” to determine the presence or absence of diabetes. Participants who responded affirmatively were classified as having diabetes. Similarly, the questionnaire “Ever told you had high blood pressure” was utilized to define hypertension, with participants answering positively beingcategorized as having hypertension.

### 2.5 Statistical analyses

Continuous variables were reported as mean ± standard deviation (SD), while categorical variables were presented as frequencies or percentages. Differences between groups were assessed using analysis of variance (ANOVA) for continuous variables and chi-square tests for categorical variables. Logistic regression was employed to calculate odds ratios (ORs) and corresponding 95% confidence intervals (CIs) for each quartile of blood cobalt levels in relation to gallstones. Three logistic regression models were developed for analysis. Model 1 did not incorporate any covariate adjustments, while Model 2 accounted for age, gender, and race. Model 3, which included comprehensive adjustments, considered age, gender, race, educational level, family income, BMI, waist circumference, serum total cholesterol level, blood selenium level, serum iron level, as well as history of diabetes and hypertension. It is important to note that some participants had missing data for certain covariates, resulting in a final sample size of 4,477 participants included in Model 3. A restricted cubic spline analysis was performed to assess the relationship between blood cobalt levels and the risk of developing gallstones. The model was adjusted for covariates including age, gender, race, educational level, family income, BMI, waist circumference. A total of 32 outliers were excluded from the model, resulting in a final sample size of 5,578 participants included in the analysis. To investigate potential variations in the relationship between blood cobalt and gallstones, subgroup analyses were performed considering factors such as age, gender, BMI, serum total cholesterol level, hypertension, and diabetes. It should be noted that there were missing covariate data for some participants, resulting in a final sample size of 5,380 participants included in the subgroup analyses. Statistical analyses were performed using R 4.2.2 software and EmpowerStats.^1^

## 3 Results

### 3.1 Baseline characteristics of participants

Table 1 presents the demographic characteristics and other covariates of the participants included in the study, categorized into quartiles based on their blood cobalt levels. Participants in Quartile 4 were found to be older compared to Quartiles 1–3. This quartile also had a higher proportion of females than males, with Non-Hispanic White participants being the majority. Furthermore, those in Quartile 4 exhibited lower BMI and waist circumference measurements, as well as lower total serum cholesterol and serum iron levels. Notably, blood selenium levels were only higher compared to the quartile with the lowest blood cobalt levels. The percentage of participants with gallstones increased wavily from Quartile 1 to Quartile 4, with Quartile 4 having the highest incidence of gallstones (15.19%, *p* < 0.001).

**Table 1:** Baseline characteristics of participants.

**Cobalt, ug/L**	**Quartile 1**	**Quartile 2**	**Quartile 3**	**Quartile 4**	***p*-value**
	[0.04, 0.11]	[0.11, 0.14]	[0.14, 0.18]	[0.18, 29.7]	
Rate of Gallstones, %	10.12%	14.12%	13.09%	15.19%	<0.001
Age,years, *M* (SD)	59.38 (11.25)	59.31 (11.10)	60.11 (11.50)	61.79 (13.03)	<0.001
Gender, %					<0.001
Male	56.96%	59.72%	48.13%	37.18%	
Female	43.04%	40.28%	51.87%	62.82%	
Race, %					<0.001
Mexican American	6.09%	9.48%	15.15%	10.84%	
Other Hispanic	13.34%	11.09%	9.29%	7.81%	
Non-Hispanic White	36.88%	37.91%	33.35%	40.58%	
Non-Hispanic Black	33.80%	27.01%	21.95%	22.81%	
Non-Hispani Asian	6.01%	9.86%	15.77%	13.74%	
Other Race	3.89%	4.64%	4.49%	4.22%	
Education, %					0.051
<9th grade	7.84%	8.53%	11.35%	8.38%	
9–11th grade	10.41%	11.56%	10.54%	12.67%	
High school graduate	25.51%	24.36%	23.82%	23.06%	
Some college or AA degree	30.72%	31.47%	31.05%	30.75%	
College graduate or above	25.51%	24.08%	23.25%	25.14%	
Ratio of family income to poverty, *M* (SD)	2.74 (1.65)	2.67 (1.62)	2.72 (1.61)	2.63 (1.62)	0.317
BMI, *M* (SD)	30.82 (7.50)	30.88 (7.22)	30.11 (6.88)	29.57 (7.24)	<0.001
Waist circumference,cm, *M* (SD)	104.37 (16.05)	104.79 (15.80)	102.60 (15.80)	101.16 (16.14)	<0.001
Total Cholesterol,mg/dL, *M* (SD)	188.05 (43.00)	189.16 (41.57)	193.07 (41.95)	186.09 (41.98)	<0.001
Selenium,ug/L, *M* (SD)	177.53 (26.82)	188.62 (27.34)	191.96 (27.26)	185.94 (30.59)	<0.001
Iron, ug/dL, *M* (SD)	87.02 (31.87)	88.78 (31.29)	89.02 (33.25)	80.61 (38.95)	<0.001
Rate of hypertension,%	49.34%	51.00%	48.50%	50.98%	0.447
Rate of diabetes,%	20.67%	22.27%	20.57%	21.17%	0.730

BMI, body mass index; *M*, mean values; SD, standard deviation.

### 3.2 Association between blood cobalt and the risk of gallstones

**Figure 2:**
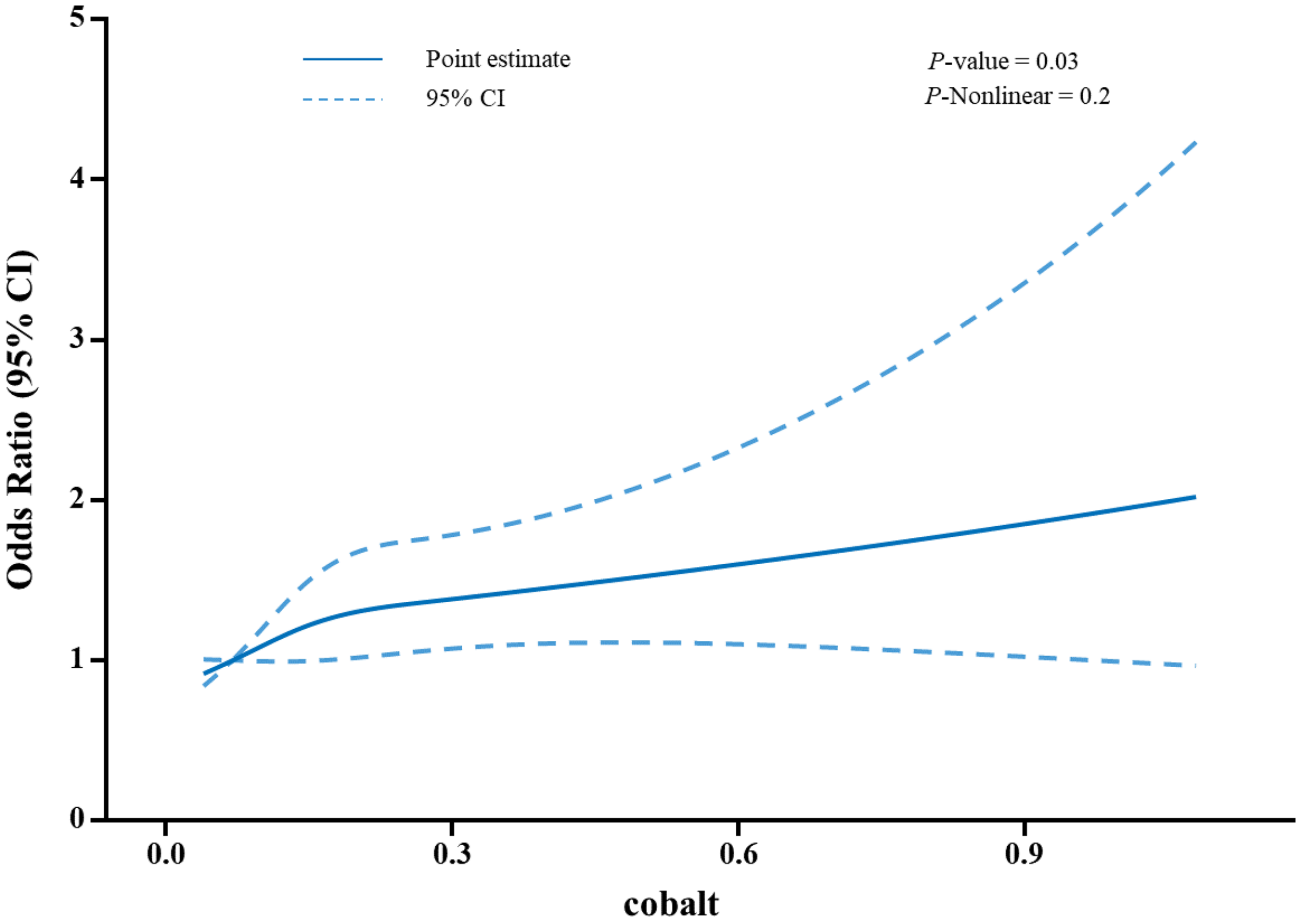
A restricted cubic spline analysis was performed to assess the relationship between blood cobalt levels and the risk of developing gallstones. The model was adjusted for covariates including age, gender, race, educational level, family income, BMI, waist circumference. A total of 32 outliers were excluded from the model, resulting in a final sample size of 5,578 participants included in the analysis. The odds ratios (OR) were plotted as a solid blue line, with 95% confidence intervals represented by the blue dashed lines.

Table 2 presents the association between blood cobalt levels and the risk of developing gallstones. For the analysis, participants were categorized into quartiles based on their blood cobalt levels. The group with the lowest concentration (Quartiles 1) was designated as the control group. In Model 1, which was the original model with no adjustment for covariates, the risk of gallstones was positively correlated with blood cobalt levels, with participants in the Quartiles 2–4 group having a 46, 34, and 59% increased risk of gallstones, respectively, compared with Quartiles 1(OR = 1.46, 95% CI: 1.14–1.87; OR = 1.34, 95% CI: 1.07–1.68; OR = 1.59, 95% CI: 1.27–1.99). In Model 2, adjustments were made for gender, age, and race. Participants in the Quartiles 2 and Quartiles 4 groups exhibited a 52 and 28% higher risk of developing gallstones, respectively, when compared to Quartiles 1(OR = 1.52, 95% CI: 1.18–1.95; OR = 1.28, 95% CI: 1.02–1.62). However, there was no significant increase in the risk of gallstones observed among participants in the Quartiles 3 group (*p* > 0.05). In Model 3, adjustments were made for multiple covariates, including age, gender, race, educational level, family income, BMI, waist circumference, serum total cholesterol level, blood selenium level, serum iron level, as well as history of diabetes and hypertension. Participants in the Quartiles 2 and Quartiles 4 groups exhibited a 54 and 35% higher risk of developing gallstones, respectively, compared to Quartiles 1(OR = 1.54, 95% CI: 1.15–2.07; OR = 1.35, 95% CI: 1.03–1.77). However, similar to Model 2, participants in the Quartiles 3 group did not show a significant increase in the risk of gallstones (*p* > 0.05). Furthermore, as illustrated in Figure 2, the restricted cubic spline analysis exhibited a positive linear correlation between blood cobalt levels and the occurrence of gallstones (*p*-Nonlinear = 0.2).

**Table 2:** Association between Cobalt and Gallstones.

**Cobalt, ug/L**		**OR (95% CI), *P*-value**	
	Model 1	Model 2	Model 3
Quartile 1, [0.04, 0.11]	1.00 (reference)	1.00 (reference)	1.00 (reference)
Quartile 2, [0.11, 0.14]	1.46 (1.14, 1.87), 0.0026	1.52 (1.18, 1.95),0.0012	1.54 (1.15, 2.07) 0.0038
Quartile 3, [0.14, 0.18]	1.34 (1.07, 1.68),0.0123	1.21 (0.96, 1.53), 0.1097	1.28 (0.98, 1.69) 0.0745
Quartile 4, [0.18, 29.7]	1.59 (1.27, 1.99),<0.0001	1.28 (1.02, 1.62),0.0344	1.35 (1.03, 1.77) 0.0312

OR, odds ratio; CI, confidence interval.

Model 1: crude model.

Model 2: adjusted for age, gender and race.

Model 3: adjusted for age, gender, race, educational level, family income, BMI, waist circumference, Serum total cholesterol level, blood selenium level, Serum iron level and a history of diabetes and hypertension.

### 3.3 Subgroup analyses

**Figure 3:**
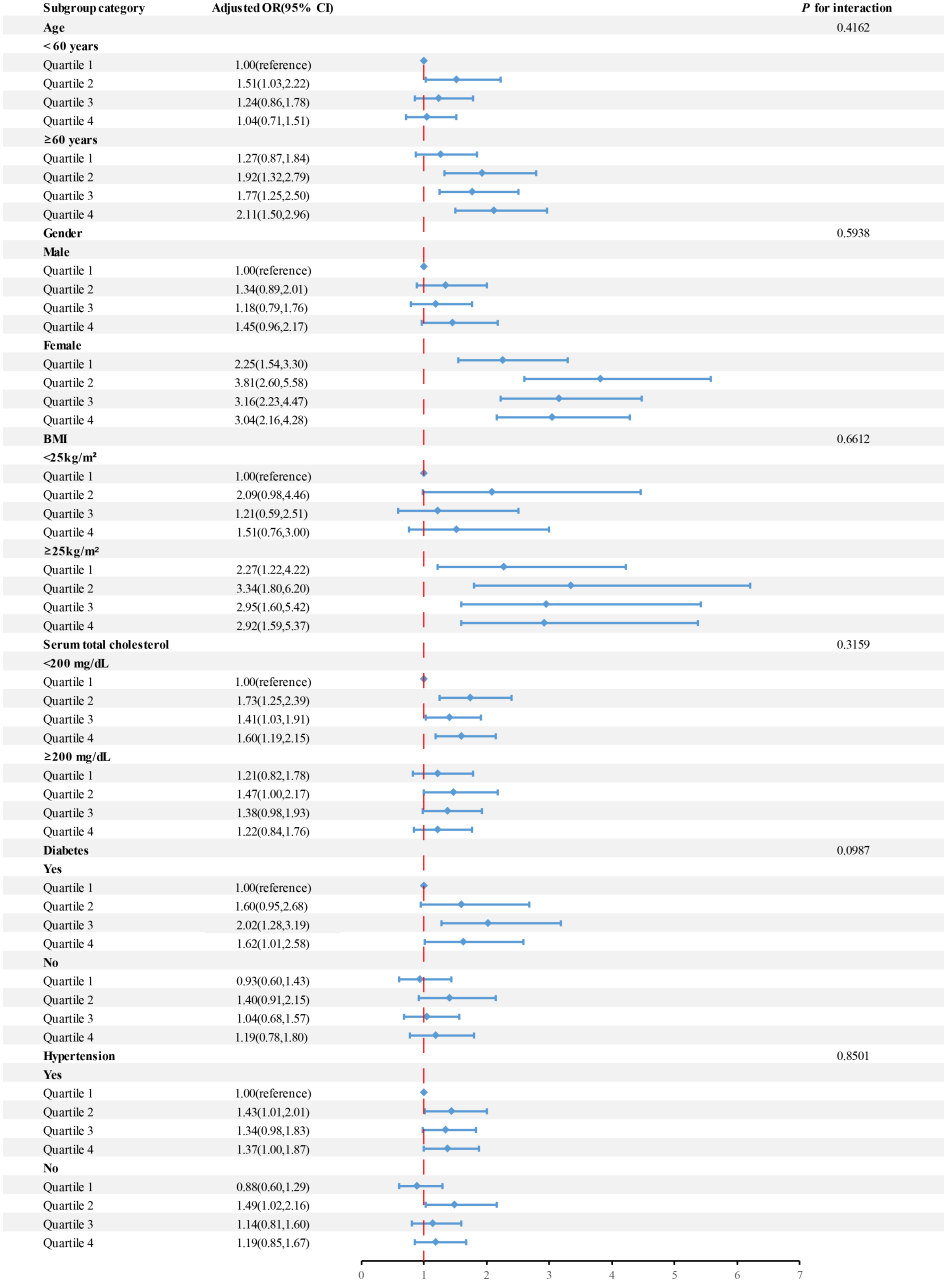
The OR and 95%CI for gallstone disease were calculated based on quartiles of cobalt levels (μg/L), with stratification by selected factors. Adjusted for age, gender, BMI, Serum total cholesterol level, and a history of diabetes and hypertension. Abbreviations: OR, odds ratio; CI, confidence interval.

Subgroup analyses were conducted to investigate whether the association between blood cobalt levels and the risk of developing gallstones was influenced by factors such as age, gender, BMI, serum total cholesterol, hypertension, and diabetes (Figure 3). After adjusting for confounding variables, individuals classified in Quartile 4 of blood cobalt levels demonstrated a significant positive correlation with an increased susceptibility to gallstones among individuals aged 60 years or older, females, those with serum cholesterol levels below 200 mg/dL, individuals with a BMI of 25 or higher, as well as those diagnosed with hypertension or diabetes.

## 4 Discussion

This cross-sectional study utilized data from the 2017–2020 NHANES to investigate the potential correlation between blood cobalt levels and the presence of gallstones. The results of this study demonstrated a noteworthy association between elevated blood cobalt levels and an increased risk of developing gallstones, even after accounting for relevant confounding factors. Notably, this association was particularly significant among individuals aged 60 years or older, females, those with serum cholesterol levels below 200 mg/dL, individuals with a BMI of 25 or higher, as well as those diagnosed with hypertension or diabetes.

It is noteworthy that no prior studies have specifically examined the correlation between blood cobalt levels and gallstones. However, our findings indicate a positive association between blood cobalt levels and the risk of developing gallstones, aligning with the research conducted by Wang et al. on the relationship between blood selenium and gallstone formation. Notably, Wang et al. also utilized data from the NHANES database and reported blood selenium as an independent risk factor for gallstones within the United States (15). Furthermore, a study conducted by Mondal et al. (16) discovered a potential link between cobalt in gallstones and an increased risk of gallbladder malignancy. Taken together, our findings highlight the significance of this study in elucidating the relationship between blood cobalt levels and gallstone risk.

The Latin term for cobalt, Cobaltum, derives from the German word “kobalt,” which translates to “subterranean devil” or “bad spirit.” This term was historically used by German miners to describe certain ores that posed harm to their health. However, contemporary understanding recognizes cobalt as an essential trace element crucial for various physiological processes. It participates in protein synthesis, contributes to myelin sheath formation in neurons, and plays a vital role in maintaining the normal functioning of the nervous system through its involvement in vitamin B12 metabolism. Cobalt’s indispensability extends to growth, development, and the overall maintenance of human health (11, 17). Nevertheless, the increasing utilization of cobalt alloys in medical prosthetic implants introduces a new source of endogenous cobalt exposure, which poses potential risks to human health (18–21). Excessive cobalt intake can lead to numerous toxic effects on the body, adversely affecting organs such as the nervous system, respiratory system, circulatory system, endocrine system, and other vital organs. Common neurological conditions associated with cobalt toxicity include neuritis, hearing impairment, and visual impairment (12, 19, 20, 22). Respiratory conditions include pneumonia, diffuse interstitial pulmonary fibrosis, and bronchial asthma (23). Cardiomyopathy is a prevalent circulatory system disorder observed in relation to cobalt exposure (24). Goiter is a common endocrine system disorder associated with excess cobalt (11). Systemic atopic dermatitis is the main skin condition linked to cobalt toxicity (25). Moreover, cobalt has the capacity to inhibit enzyme activity, compromise the body’s immune response, and even contribute to carcinogenesis. As a result, the International Agency for Research on Cancer (IARC) has classified cobalt and its compounds as probable carcinogens (26).

The mechanisms responsible for the transport of cobalt into mammalian cells remain insufficiently understood. However, it has been established that the divalent metal ion transporter 1(DMT1) as well as the Zip-8 and Zip-14 transporters play significant roles in facilitating the penetration of cobalt and other heavy elements across tissue barriers and into cells (27). Cobalt triggers the onset of oxidative stress, resulting in cytotoxicity and cell death. It induces the excessive production of oxygen radicals, which catalyze the formation of highly toxic hydroxyl radicals while concurrently inhibiting cellular antioxidant capacity. Consequently, this process disrupts the balance between cellular oxidation and antioxidant defenses. Oxygen free radicals inflict damage to cells through various mechanisms including DNA damage, protein modification, stimulation of oncogenic gene expression, and activation of nuclear transcription factors (26, 28–30). In rat liver, Co^2+^ has demonstrated the ability to induce a shift in mitochondrial permeability, causing mitochondrial swelling and collapse of the membrane potential. Furthermore, Co^2+^ triggers the release of pro-apoptotic factors and cytochrome c, ultimately inducing mitochondrial oxidative stress-induced apoptosis. On the other hand, Co^2+^ can impede the degradation of hypoxia-inducible factor (HIF) by hydroxylase, leading to cellular hypoxia and the subsequent development of irreversible oxygen deprivation (17). We postulate that cobalt-induced damage to hepatocytes and intracellular cells within the gallbladder may contribute to disorders in cholesterol metabolism and gallbladder motility, thereby elevating the risk of gallstone formation in conjunction with increased blood cobalt levels. However, further basic experimental studies are required to elucidate the specific underlying mechanisms. In Model 2 and 3, there was no observed increase in the risk of gallstones among those in Quartiles 3 compared to Quartiles 1. This discrepancy may be attributed to the inclusion of covariates in the analyses.

Subgroup analysis revealed population-specific variations in blood cobalt levels and the risk of gallstones. Notably, older individuals aged ≥60 years exhibited heightened susceptibility to cobalt exposure compared to those under 60 years old. This observation may be attributed to the increased likelihood of older individuals utilizing medically derived cobalt-alloyed prostheses such as hip and knee implants (14, 31, 32). Obesity serves as a predisposing factor for gallstone formation, while estrogen enhances the risk by promoting hepatic cholesterol synthesis and secretion while inhibiting bile salt synthesis. Furthermore, high blood pressure, insulin resistance, and diabetes independently contribute to gallstone development (1, 33–37). These factors likely explain the association between elevated blood cobalt levels and an increased risk of gallstones among women, obese individuals, and patients with hypertension or diabetes. The relationship between serum cholesterol levels and gallstone occurrence has yielded conflicting findings. Some studies by Shinchi K, Duque MX, Attili, A. F et al. indicated that low cholesterol levels increased the risk of gallstones (38–40); Conversely, research conducted by Jiwen Wang, I. N Grigor’eva et al. demonstrated that higher cholesterol levels were associated with an increased risk (41, 42). However, S Kono’s study did not identify a significant association between serum cholesterol levels and gallstones (43). Our study revealed a significant association between elevated blood cobalt levels and an increased risk of gallstones, particularly among individuals with low serum cholesterol levels. However, further investigation is warranted to delve into the precise mechanisms that underlie this observation. Future studies should consider variations in study design, study populations, and methods of serum cholesterol measurement to gain a comprehensive understanding of this relationship.

The study has the following strengths. First, the study is an important guide to further preventing and reducing the incidence of gallstones among Americans. Second, the data for the study was obtained from NHANES, which used a nationally representative sample and a standardized experimental testing protocol to effectively reduce error in the study. This choice ensured the representativeness of the study and made the experimental results more reliable. This study has several limitations that should be acknowledged. Firstly, due to its cross-sectional design, it was not possible to establish a causal relationship between high blood cobalt levels and an increased risk of gallstones. Additionally, the measurement of blood cobalt levels was conducted only once, which may not provide an accurate representation of an individual’s long-term cobalt exposure or fluctuations in their blood cobalt levels over time. These limitations highlight the need for future studies with longitudinal designs and repeated measurements to better understand the association between blood cobalt levels and the risk of gallstones.

In conclusion, our study findings indicate a notable association between elevated blood cobalt levels and an increased risk of gallstones. This relationship holds particular significance in the following subgroups: individuals aged >60 years, women, those with serum cholesterol levels <200 mg/dL, BMI >25, and individuals with hypertension or diabetes.

## Data availability statement

The dataset, which supports the conclusions drawn in this paper, is accessible through the NHANES repository at https://www.cdc.gov/nchs/nhanes/index.htm.

## Ethics statement

The studies involving humans were approved by the Ethical Review Board of the National Center for Health Statistics. The studies were conducted in accordance with the local legislation and institutional requirements. Written informed consent for participation in this study was provided by the participants’ legal guardians/next of kin.

## Author contributions

YL: Data curation, Methodology, Validation, Writing – original draft, Writing – review & editing. HH: Investigation, Software, Writing – original draft, Writing – review & editing. KY: Investigation, Software, Writing – original draft. CM: Investigation, Software, Writing – original draft. XF: Formal analysis, Funding acquisition, Investigation, Software, Validation, Writing – original draft, Writing – review & editing.

## Glossary

**Table:** 

BMI	Body mass index
M	Mean values
SD	Standard deviation
OR	Odds ratio
CI	confidence interval
NHANES	National health and nutrition examination survey
ICP-MS	Inductively coupled plasma mass spectrometry
NCHS	National center for health statistics
LLOD	Lower limit of detection
IARC	International Agency for Research on Cancer
DMT1	Divalent metal ion transporter 1
HIF	hypoxia-inducible factor
Co	Cobalt

## Footnotes

^1^ http://www.empowerstats.com
